# Construction of Monolayer Ti_3_C_2_T_x_ MXene on Nickel Foam under High Electrostatic Fields for High-Performance Supercapacitors

**DOI:** 10.3390/nano14100887

**Published:** 2024-05-19

**Authors:** Liyong Zhang, Jijie Chen, Guangzhi Wei, Han Li, Guanbo Wang, Tongjie Li, Juan Wang, Yehu Jiang, Le Bao, Yongxing Zhang

**Affiliations:** 1 Department of Mechanical Engineering, Anhui Science and Technology University, Chuzhou 235000, China; 2 Anhui Province Key Laboratory of Pollutant Sensitive Materials and Environmental Remediation, Anhui Province Key Laboratory of Intelligent Computing and Applications, Anhui Province Industrial Generic Technology Research Center for Alumics Materials, Huaibei Normal University, Huaibei 235000, China; 3 Jiangsu Zhonggong High-End Equipment Research Institute Co., Ltd., Taizhou 235000, China; 4 Anhui Zhongxin Technology Co., Ltd., Chuzhou 235000, China; 5 Department of Mechatronics Engineering, Hanyang University, Ansan 15588, Republic of Korea; baole@hanyang.ac.kr

**Keywords:** monolayer Ti_3_C_2_T_x_ MXene, high electrostatic fields, electrostatic spray deposition, self-supporting electrode, supercapacitor

## Abstract

Ti_3_C_2_T_x_ MXene, as a common two-dimensional material, has a wide range of applications in electrochemical energy storage. However, the surface forces of few-layer or monolayer Ti_3_C_2_T_x_ MXene lead to easy agglomeration, which hinders the demonstration of its performance due to the characteristics of layered materials. Herein, we report a facile method for preparing monolayer Ti_3_C_2_T_x_ MXene on nickel foam to achieve a self-supporting structure for supercapacitor electrodes under high electrostatic fields. Moreover, the specific capacitance varies with the deposition of different-concentration monolayer Ti_3_C_2_T_x_ MXene on nickel foam. As a result, Ti_3_C_2_T_x_/NF has a high specific capacitance of 319 mF cm^−2^ at 2 mA cm^−2^ and an excellent long-term cycling stability of 94.4% after 7000 cycles. It was observed that the areal specific capacitance increases, whereas the mass specific capacitance decreases with the increasing loading mass. Attributable to the effect of the high electrostatic field, the self-supporting structure of the Ti_3_C_2_T_x_/NF becomes denser as the concentration of the monolayer Ti_3_C_2_T_x_ MXene ink increases, ultimately affecting its electrochemical performance. This work provides a simple way to overcome the agglomeration problem of few-layer or monolayer MXene, then form a self-supporting electrode exhibiting excellent electrochemical performance.

## 1. Introduction

With the rapid development of electronic devices, the demand for portable energy storage devices is steadily increasing [1]. Supercapacitors, recognized for their high power density and rapid charge–discharge capabilities, have attracted extensive research interest [2,3,4]. Moreover, electrode materials play a pivotal role in the performance of supercapacitor devices [5,6]. Therefore, the development of high-performance electrode materials is crucial to realize the ultimate goal of making supercapacitors practical and efficient.

Two-dimensional (2D) materials are extensively researched due to their high specific surface area, outstanding electrical properties, and tunable interlayer characteristics [7,8,9]. There are various 2D materials, including graphene, transition metal dichalcogenides (TMDs), black scales, transition metal carbides and nitrides (MXenes), and so on [10]. MXenes are produced through the selective etching of the original MAX phase materials by hydrofluoric acid (HF), defined by a chemical formula of M_n+1_AX_n_, where n can be 1, 2, or 3 (M_2_AX, M_3_AX_2_, or M_4_AX_3_) [11]. MXene, as a highly attractive electrode material for supercapacitors, possesses several advantageous properties, like its excellent conductivity, high specific surface area, abundant functional groups, noticeable pseudocapacitance working mechanism, and good hydrophilicity [12,13,14].

To further enhance the electrochemical performance of MXene, various strategies are currently being implemented, such as surface modification, doping impurity atoms, microstructure control, heterostructure preparation with other materials, and monolayer treatment [15]. A reduction in interlayer spacing and ion diffusion can be directly achieved through monolayer treatment [16]. Larger interlayer spacing allows for greater ion intercalation or deintercalation in the electrolyte, while short-distance ion diffusion can enhance ion diffusion kinetics [17,18,19]. Over the past decade, several strategies, including mechanical exfoliation and lithium ion intercalation, have been developed to obtain single-layer or few-layer MXene from bulk sources [20,21,22]. Unfortunately, exfoliated MXene layers tend to self-aggregate during operation, leading to the loss of their electrochemical benefits and, ultimately, resulting in low specific capacitance, which significantly limits their standalone application [23,24]. Therefore, combining single-layer or few-layer MXene with other substrate materials as a scaffold can help overcome the aggregation issue associated with layered materials.

Ti_3_C_2_T_x_ is one of the common types of MXenes, being distinguished by its chemical stability, and it is commonly employed as an electrode material for supercapacitors [25,26,27]. Ti_3_C_2_T_x_ is obtained by the HF etching of Ti_3_AlC_2_, where T represents the surface functional groups (-OH/-F/-O) [28,29,30]. Ti_3_C_2_T_x_ MXene has excellent conductivity, which is beneficial for improving the performance of supercapacitors [31]. In recent years, many studies have been conducted on Ti_3_C_2_T_x_ MXene as an electrode material for supercapacitors. Hu and co-workers prepared d-Ti_3_C_2_T_x_ MXene, which displays a high specific capacitance of 400 F g^−1^ at 2 mV s^−1^ [32]. Kayali and colleagues synthesized Ti_3_C_2_T_x_ MXene, showing a specific capacitance of 435 F g^−1^ at 2 mV s^−1^ [11]. Yu et al. prepared AC/Ti_3_C_2_T_x_ as a supercapacitor electrode, exhibiting a specific capacitance of 126 F g^−1^ at 0.1 A g^−1^ [33]. Pathak et al. synthesized a Ti_3_C_2_T_x_ MXene-decorated porous carbon nanofiber which shows a high specific capacitance of 572.7 F g^−1^ at 1 A g^−1^ [34].

Herein, an effective method involving the enrichment of monolayer Ti_3_C_2_T_x_ MXene on nickel foam (NF) through electrostatic spray deposition (ESD) is reported. This method prevents aggregation, exposes more active sites, and produces a self-supporting electrode that displays the intrinsic characteristics of electrode materials. Monolayer Ti_3_C_2_T_x_ MXene at varying concentrations was deposited on NF, and the supercapacitor performance was evaluated. The Ti_3_C_2_T_x_/NF-2.0 electrode exhibited a high specific capacitance of 319 mF cm^−2^ at 2 mA cm^−2^ and a superior cycling stability of 94.4% after 7000 cycles. Furthermore, the relationship between loading mass and specific capacitance was investigated. This operational strategy effectively addresses the issue of aggregation in layered materials to achieve enhanced specific capacitance.

## 2. Materials and Methods

### 2.1. Preparation of Ti_3_C_2_T_x_ MXene

The Ti powder, Al powder, and C powder were mixed in a ratio of 3:1.1:2 and ball-milled for 1 h to achieve a uniformly mixed powder. Subsequently, the obtained powder was compressed into cylindrical particles with a 13 mm diameter under a pressure of 1 GPa. These particles were then subjected to a gradual heating process in a tube furnace, starting at a rate of 9 °C/min up to 1000 °C, followed by further heating to 1400 °C at a rate of 5 °C/min for 2 h under a continuous flow of argon. Upon cooling to room temperature, the sample was manually ground and crushed into powder. Slowly adding 40% HF to the sample, the mixture was stirred for 24 h. In the case of centrifugation, the sample underwent repeated washing with deionized water until the pH value of the supernatant exceeded 6. Finally, the resulting black product was placed in a vacuum drying oven for 12 h.

### 2.2. Preparation of Ti_3_C_2_T_x_ MXene Ink

The sediment was re-dispersed in 200 mL of deionized water and bath-sonicated (Shanghai Kedao Ultrasonic Instrument Co., Ltd., Shanghai, China, model SK5200HP, 53 kHz) under argon bubbling in an ice water bath for 1 h. A stable Ti_3_C_2_T_x_ dispersion was obtained by collecting the top 80% supernatant after centrifugation at 3500 rpm for 30 min. An appropriate amount of the Ti_3_C_2_T_x_ MXene dispersion underwent further centrifugation at 5000 rpm for an additional 20 min. The resulting sediment was then collected and re-dispersed in 10 mL of deionized water through vigorous hand shaking for 15 min, yielding a viscous Ti_3_C_2_T_x_ MXene ink.

### 2.3. Preparation of Ti_3_C_2_T_x_/NF

Ti_3_C_2_T_x_ on nickel foam (NF) (Ti_3_C_2_T_x_/NF) was successfully obtained using electrostatic spray deposition (ESD) technology under high potentials (6–9 kV) in open air.

### 2.4. Material Characterizations

XRD spectra were acquired using a Philips X′pert PRO X-ray diffractometer with Cu K radiation (λ = 0.154 nm). X-ray Photoelectron Spectroscopy (XPS, Thermo ESCALAB 250Xi, Breda, The Netherlands) was carried out with a monochromatic Al Kα source at 1486.6 eV. Nanostructure characterizations for the materials were carried out using Field Emission Scanning Electron Microscopy (FE-SEM, Quanta 200FEG, Peabody, MA, USA), Energy-Dispersive X-ray Spectroscopy (EDS, Oxford EDS with INCA software INCA V7.5), and Transmission Electron Microscopy (TEM, JEM-2100, Tokyo, Japan).

### 2.5. Electrochemical Measurement

Electrochemical data were generated using the Dutch Ivium (Vertex.C.DC) electrochemical station in 1 M Na_2_SO_4_ electrolyte, allowing for the acquisition of cyclic voltammetry (CV), galvanostatic charge–discharge (GCD), and electrochemical impedance spectroscopy (EIS) measurements. EIS was conducted by applying an open-circuit potential with an amplitude of 5 mV across a frequency range from 100 kHz to 0.01 Hz. The three-electrode system consists of a counter electrode, a reference electrode, and a working electrode. Among them, the 1 × 1 cm^−2^ platinum foil electrode serves as the counter electrode, Ag/AgCl (in 1 M KCl) is used as the reference electrode, and the prepared sample acts as the working electrode.

## 3. Results and Discussion

**Figure 1:**
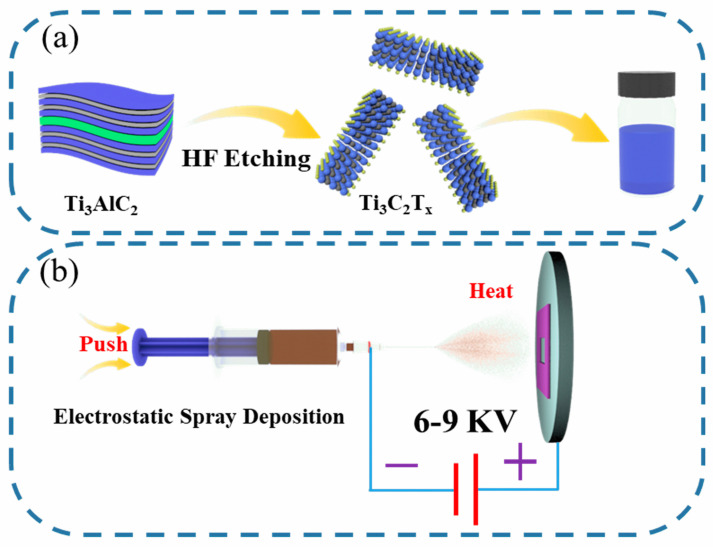
(**a**,**b**) Schematic diagram of preparation of monolayer Ti_3_C_2_T_x_ MXene ink and self-supporting electrode of Ti_3_C_2_T_x_/NF.

As shown in Figure 1a, the monolayer Ti_3_C_2_T_x_ MXene was successfully synthesized through solid-phase reaction, HF treatment, and bath sonication. As shown in Figure 1b, Ti_3_C_2_T_x_/NF electrodes can be prepared by depositing viscous Ti_3_C_2_T_x_ MXene ink under the assistance of high electrostatic fields (6–9 kV). Different self-supporting electrodes can be prepared by varying the concentrations of monolayer Ti_3_C_2_T_x_ MXene ink. The ones constructed in this study are named Ti_3_C_2_T_x_/NF-1.0, Ti_3_C_2_T_x_/NF-2.0, and Ti_3_C_2_T_x_/NF-3.0.

**Figure 2:**
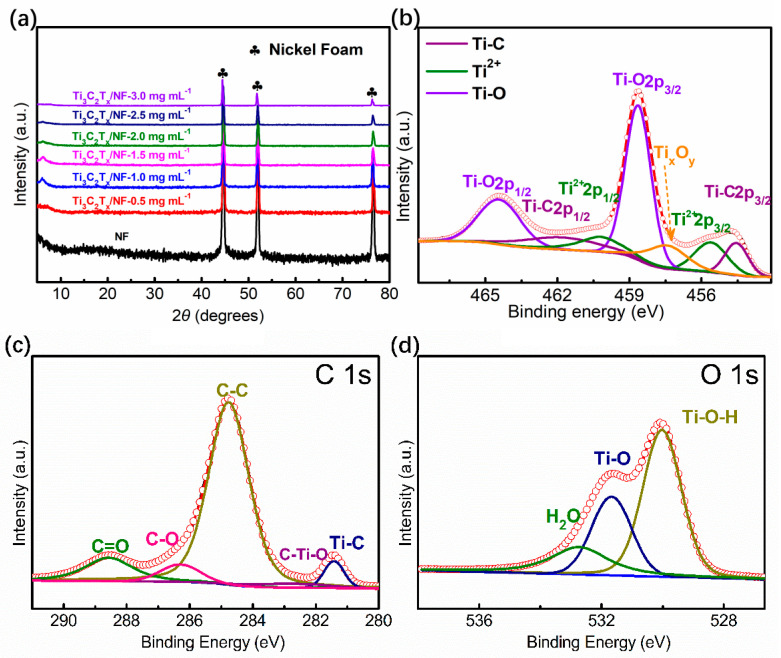
(**a**) XRD patterns of Ti_3_C_2_T_x_/NF-1.0, Ti_3_C_2_T_x_/NF-2.0, and Ti_3_C_2_T_x_/NF-3.0 electrodes and NF. (**b**–**d**) Deconvolution of Mo 3d, C 1s, and O 1s spectra for Ti_3_C_2_T_x_/NF-2.0 electrode.

To verify the crystal structure of all the samples, X-ray diffraction (XRD) was applied. From Figure 2a, the (002) peaks of the Ti_3_C_2_T_x_/NF-1.0, Ti_3_C_2_T_x_/NF-2.0, and Ti_3_C_2_T_x_/NF-3.0 electrodes are located at 6.2°; the interlayer spacing is 14.2 nm. The wide interlayer spacing not only allows for more ions to be intercalated or deintercalated but also effectively shortens the diffusion path of the ions. Obviously, the intensity of the (002) peak gradually decreases with the increasing concentrations of monolayer Ti_3_C_2_T_x_ MXene ink, owing to the significant internal strain. It is clear that the peaks located at 44.6°, 51.8° and 76.3° represent the characteristic peaks of NF as an excellent and common collector [35]. In addition, the intensity of these peaks also gradually decreases, which indicates that the structure becomes denser.

Information regarding element valence state was collected by X-ray Photoelectron Spectroscopy (XPS). As shown in Figure 2b, for the high-resolution Ti 2p spectrum of Ti_3_C_2_T_x_/NF-2.0 electrode, some peaks are located at 461.8, 454.5, 464.4, and 458.6 eV. Firstly, the former two represent the Ti-C bond of Ti_3_C_2_T_x_, reflecting the integrity of the crystal structure. Secondly, the latter two correspond to the Ti-O bond. In addition, the peak shown at a lower bonding energy of 457.4 eV is related to the Ti ion with the bond state of Ti_x_O_y_, suggesting the formation of TiO_2_ with oxidation because of monolayer Ti_3_C_2_T_x_ MXene being prone to oxidation in air. From the high-resolution C 1s and O 1s spectra in Figure 2c,d, the bonding of C-Ti-O, Ti-C, Ti-O, and Ti-O-H can be seen clearly, reflecting the stable existence of monolayer Ti_3_C_2_T_x_ MXene after electrostatic spray deposition on NF.

**Figure 3:**
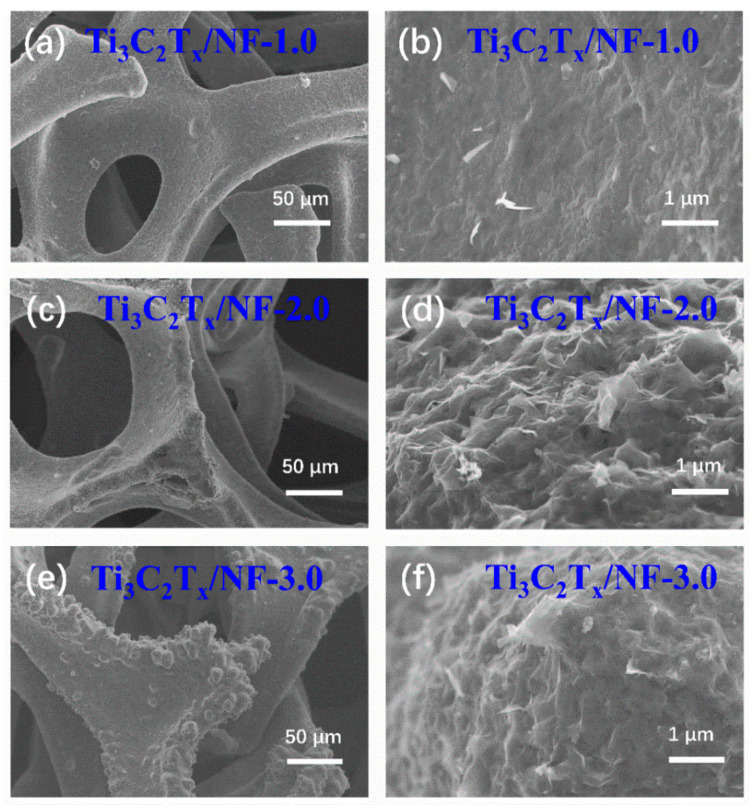
(**a**–**f**) SEM images of Ti_3_C_2_T_x_/NF-1.0, Ti_3_C_2_T_x_/NF-2.0, and Ti_3_C_2_T_x_/NF-3.0 electrodes.

The morphology of all samples was observed by Scanning Electron Microscopy (SEM). As shown in Figure 3, monolayer Ti_3_C_2_T_x_ MXene is uniformly deposited on the NF substrate under the assistance of high electrostatic fields to form a self-supporting structure, effectively combining active materials with the collector through electrostatic force. NF, a porous framework substrate, not only provides high conductivity but also promotes contact between the electrode materials and the electrolyte [36,37]. Furthermore, the self-supporting structure formed has more voids when the concentration of monolayer Ti_3_C_2_T_x_ MXene ink is low, allowing for more ions to move in and out of the electrolyte. In contrast, the self-supporting structure becomes denser and more stable as the concentration of Ti_3_C_2_T_x_ MXene ink gradually increases.

**Figure 4:**
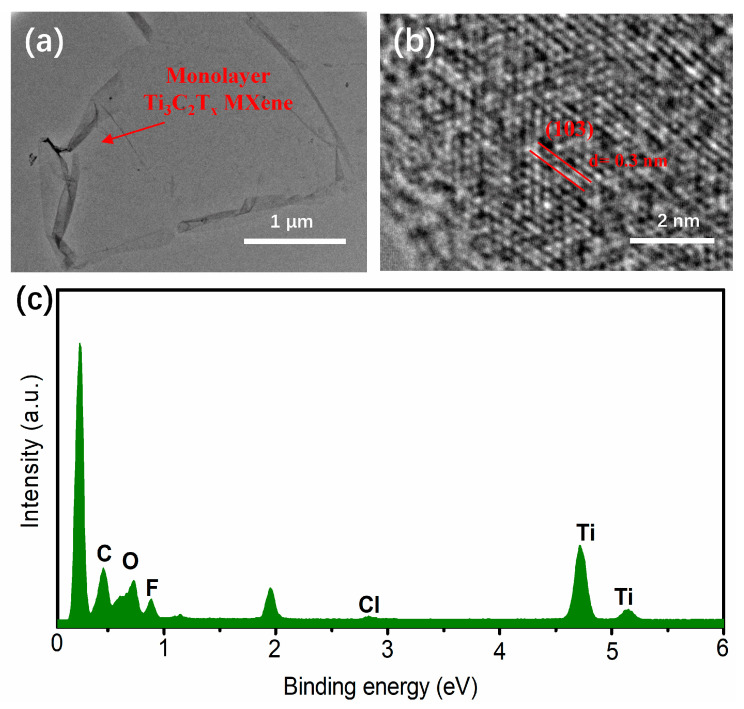
(**a**–**c**) TEM image, HRTEM image, and EDX result of monolayer Ti_3_C_2_T_x_ MXene.

The microstructure of Ti_3_C_2_T_x_ MXene was studied by Transmission Electron Microscopy (TEM). From Figure 4a, it is obvious that a monolayer structure is presented, suggesting the successful preparation of monolayer Ti_3_C_2_T_x_ MXene. As shown in Figure 4b, there is one visible interlayer spacing of 0.3 nm in the single-layer structure, which is consistent with the (103) lattice plane of hexagonal Ti_3_C_2_T_x_ MXene, confirming the successful preparation of monolayer Ti_3_C_2_T_x_ MXene again. As illustrated in Figure 4c, the Energy Dispersive X-ray (EDX) spectrum confirms the presence of elements Ti, C, O, and Cl on the Ti_3_C_2_T_x_ MXene.

**Figure 5:**
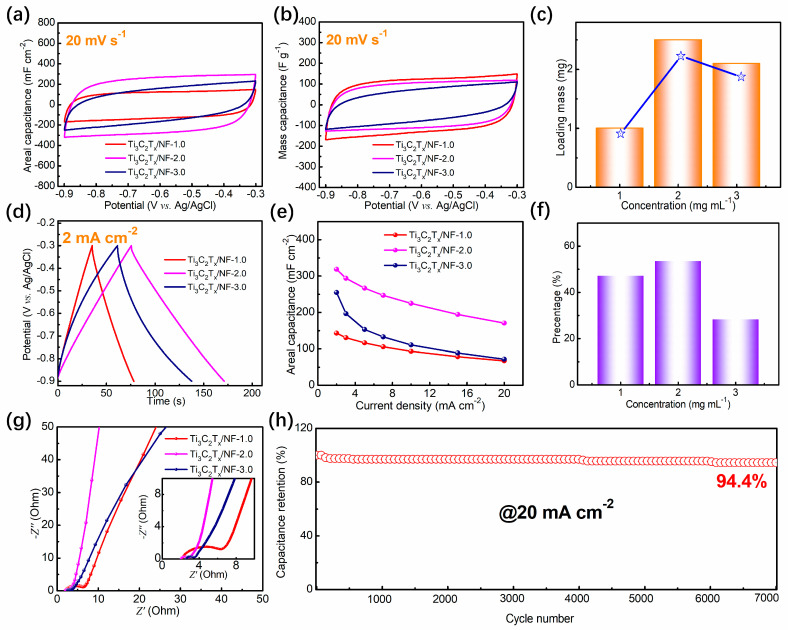
(**a**) The cycle voltammogram (CV) curves of the Ti_3_C_2_T_x_/NF-1.0, Ti_3_C_2_T_x_/NF-2.0, and Ti_3_C_2_T_x_/NF-3.0 electrodes for areal specific capacitance at 20 mV s^−1^. (**b**) The CV curves of the Ti_3_C_2_T_x_/NF-1.0, Ti_3_C_2_T_x_/NF-2.0, and Ti_3_C_2_T_x_/NF-3.0 electrodes for mass specific capacitance at 20 mV s^−1^. (**c**) The loading mass of the Ti_3_C_2_T_x_/NF-1.0, Ti_3_C_2_T_x_/NF-2.0, and Ti_3_C_2_T_x_/NF-3.0 electrodes. (**d**) The galvanostatic charge–discharge (GCD) curves of the Ti_3_C_2_T_x_/NF-1.0, Ti_3_C_2_T_x_/NF-2.0, and Ti_3_C_2_T_x_/NF-3.0 electrodes at 2 mA cm^−2^. (**e**,**f**) The performance rates of the Ti_3_C_2_T_x_/NF-1.0, Ti_3_C_2_T_x_/NF-2.0, and Ti_3_C_2_T_x_/NF-3.0 electrodes. (**g**) The electrochemical impedance spectroscopy (EIS) curves of the Ti_3_C_2_T_x_/NF-1.0, Ti_3_C_2_T_x_/NF-2.0, and Ti_3_C_2_T_x_/NF-3.0 electrodes. (**h**) The capacitance retention of Ti_3_C_2_T_x_/NF-2.0 after 7000 cycles at 20 mA cm^−2^.

To evaluate the supercapacitor performance of the electrodes, the cycle voltammogram (CV), galvanostatic charge–discharge (GCD), and electrochemical impedance spectroscopy (EIS) curves were all tested. As shown in Figure 5a, the potential windows are from −0.9 to −0.3 V for the Ti_3_C_2_T_x_/NF-1.0, Ti_3_C_2_T_x_/NF-2.0, and Ti_3_C_2_T_x_/NF-3.0 electrodes. In addition, the areal surrounded by CV curves represents the specific capacitance for each electrode. The Ti_3_C_2_T_x_/NF-2.0 electrode possesses the biggest areal, which indicates it has a higher areal specific capacitance than the other two electrodes. As shown in Figure 5b, the Ti_3_C_2_T_x_/NF -3.0 electrode has the biggest areal, suggesting it has the highest mass specific capacitance. According to Figure 5c, the loading masses of Ti_3_C_2_T_x_/NF-1.0, Ti_3_C_2_T_x_/NF-2.0, and Ti_3_C_2_T_x_/NF-3.0 are 1, 2.5, and 2.1 mg, respectively. When the concentration of monolayer Ti_3_C_2_T_x_ MXene ink is low, the loading mass gradually increases under the effect of the electrostatic force. However, the loading mass reaches a certain threshold at high concentrations. As a result, the areal specific capacitance, as shown in Table 1, increases first and then decreases, while the mass specific capacitance gradually decreases at the same scan rate of 20 mV s^−1^ with the increase in loading mass. This is mainly determined by the self-supporting structure after ESD. On the one hand, the areal specific capacitance is affected by the loading mass on the electrodes. On the other hand, the areal specific capacitance and mass specific capacitance are affected by the structure of the active substances on the electrodes. In Figure 5d, the areal specific capacitances of all electrodes at a current density of 2 mA cm^−2^ are displayed in the form of GCD curves; we calculated values of 143, 319, and 255 mF cm^−2^, respectively. Additionally, the areal specific capacitances of the Ti_3_C_2_T_x_/NF-2.0 electrode, shown in Figure 5e for the same current densities of 2, 3, 5, 7, 10, 15, and 20 mA cm^−2^, are higher than those of the Ti_3_C_2_T_x_/NF-1.0 and Ti_3_C_2_T_x_/NF-3.0 electrodes, indicating the excellent supercapacitor performance for Ti_3_C_2_T_x_/NF-2.0 electrode. In addition, the performance rates of the Ti_3_C_2_T_x_/NF-1.0, Ti_3_C_2_T_x_/NF-2.0, and Ti_3_C_2_T_x_/NF-3.0 electrodes, as shown in Figure 5f, are 47.1%, 53.5%, and 28.3%, respectively.

EIS is also a crucial parameter reflecting the electrochemical performance of the material. In EIS data, high frequency and low frequency represent different electrochemical processes. The high-frequency range typically corresponds to charge transfer resistance (*R_ct_*), indicated by the diameter of a semicircle and ion migration in the electrolyte and equivalent series resistance (ESR), while the low-frequency range usually corresponds to the double-layer capacitance on the electrode surface. After fitting, it was found that the *R_ct_* values of Ti_3_C_2_T_x_/NF-1.0, Ti_3_C_2_T_x_/NF-2.0, and Ti_3_C_2_T_x_/NF-3.0 are 6.1, 4.2, and 2.2 Ω, respectively. Obviously, *R_ct_* decreases with the increase in the concentration of monolayer Ti_3_C_2_T_x_ MXene ink, demonstrating that Ti_3_C_2_T_x_ MXene has high conductivity. Additionally, the Ti_3_C_2_T_x_/NF-2.0 electrode holds a small ESR, which benefits the adsorption of Na+ ions in the porous structure.

Long-term cycle measurement at a high current density is of great significance for supercapacitors. The performance stability and lifespan characteristics of supercapacitors under high loads and prolonged operation can be evaluated, providing important reference and validation for practical applications. Figure 5h demonstrates the cycling stability of the Ti_3_C_2_T_x_/NF-2.0 electrode. After 7000 charge–discharge cycles at 20 mA cm^−2^, the self-supporting electrode can still retain 94.4% of the initial capacitance, which indicates excellent cycling stability.

**Figure 6:**
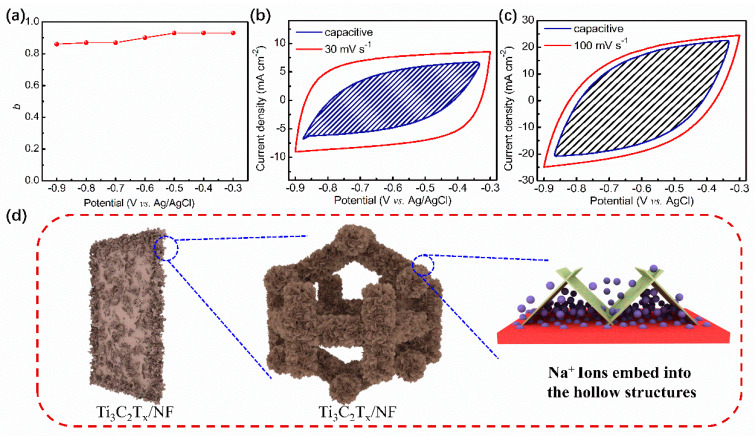
(**a**) *b*-value for the Ti_3_C_2_T_x_/NF-2.0 electrode. (**b**,**c**) CV partition analysis showing the capacitive contribution to the total current at select scan rates of 30 and 100 mV s^−1^. (**d**) Na^+^ ion diffusion mechanism diagram for the Ti_3_C_2_T_x_/NF-2.0 electrode.

In Figure S1, the CV and GCD curves of all the as-prepared electrodes show nearly rectangular and triangular shapes, demonstrating capacitive electrochemical behaviors. To ascertain the accuracy of this finding, the value of *b* of all electrodes was calculated by fitting. Furthermore, charge storage mechanisms were studied for the Ti_3_C_2_T_x_/NF electrodes. The relationship between the current at the potential (*I*(*V*)) and the scan rate (*v*) is suggested to be as follows: *I*(*V*) = *av^b^*. Here, *a* and *b* represent the constant, and *v* represents the scan rate. In electrochemical research, for the *b*-value fitting calculation, the value close to 0.5 indicates that the electrochemical process is diffusion-controlled, while the value close to 1 indicates that the electrode material has capacitive-like behavior [38,39,40]. In Figure 6a and Figure S2, the *b*-values of the Ti_3_C_2_T_x_/NF-1.0 and Ti_3_C_2_T_x_/NF-2.0 electrodes range from 0.78 to 0.97, suggesting capacitive-like behavior. However, the *b*-value of the Ti_3_C_2_T_x_/NF-3.0 electrode ranges from 0.65 to 0.77, which results from the disappearance of porous self-supporting structures caused by high concentrations of monolayer Ti_3_C_2_T_x_ MXene ink and the oxidation of high concentrations of monolayer Ti_3_C_2_T_x_ MXene ink.

Calculating the capacitance contribution helps to evaluate the relative importance of capacitance contribution compared to other electrochemical processes, such as diffusion control, thus providing a better understanding of the electrochemical performance of materials. The contribution of capacitance and diffusion limitations to the total capacitance can be further quantified by the following equation: *i*(*V*) = *k*_1_*v* + *k*_2_*v*^1/2^. Here, *k*_1_*v* represents the capacitance contribution, while *k*_2_*v*^1/2^ represents the diffusion contribution [41,42]. As shown in Figure S3 and Figure 6b,c, the capacitance contribution of the Ti_3_C_2_T_x_/NF-1.0 electrode increased from 72% at 30 mV s^−1^ to 91% at 100 mV s^−1^. The capacitance contribution of the Ti_3_C_2_T_x_/NF-2.0 electrode increased from 60% to 78%, while the capacitance contribution of the Ti_3_C_2_T_x_/NF electrodes increased from 42% to 59%. As shown in Figure 6d, this result is consistent with the calculated *b*-values. The low concentrations of monolayer Ti_3_C_2_T_x_ MXene ink tend to help form porous structures under the effect of the high electrostatic fields, thereby increasing the capacitance contribution. Conversely, the high concentrations of monolayer Ti_3_C_2_T_x_ MXene ink result in the formation of dense self-supporting structures under the effect of the high electrostatic fields, leading to oxidation reactions and the generation of TiO_2_.

To demonstrate the applicability of the Ti_3_C_2_T_x_/NF electrodes, a symmetrical device was assembled using two Ti_3_C_2_T_x_/NF electrodes. The CV and GCD curves are shown in Figure S4, and they were obtained by testing the supercapacitor performance. It can be observed by viewing the CV curves that the potential window of the device is 0.6 V in Figure S5. Furthermore, the specific capacitance of the device, based on the GCD curves, is 201.5 mF cm^−2^ at 0.3 mA cm^−2^. The *b*-value of the symmetric supercapacitor device has a range from 0.7 to 0.8, indicating capacitor-like behavior. The assembled symmetric supercapacitor device exhibits a high energy density of 10 μWh cm^−2^ and a power density of 630 μWh cm^−2^, values which are better than most of the previously reported devices, as shown in Figure S6.

## 4. Conclusions

A self-supporting Ti_3_C_2_T_x_/NF electrode structure was successfully prepared by using monolayer Ti_3_C_2_T_x_ MXene ink under high electrostatic fields. This strategy can effectively suppress the aggregation of monolayer Ti_3_C_2_T_x_ MXene, thereby exhibiting excellent supercapacitor performance, as evidenced by a high specific capacitance of 319 mF cm^−2^ at 2 mA cm^−2^ and an excellent long-term cycling stability of 94.4% after 7000 cycles. Furthermore, the different electrochemical properties of the self-supporting structure obtained by the electrostatic spray deposition of monolayer Ti_3_C_2_T_x_ MXene with varying concentrations were investigated, and the underlying reasons were analyzed. This method provides a reference for the preparation of self-supporting electrodes with low aggregation and high-performance layered materials.

## Figures and Tables

**Table 1:** The loading mass, areal capacitance, and mass capacitance values of the Ti_3_C_2_T_x_/NF-1.0, Ti_3_C_2_T_x_/NF-2.0, and Ti_3_C_2_T_x_/NF-3.0 electrodes.

	1 mg mL^−1^	2 mg mL^−1^	3 mg mL^−1^
Loading mass	1 mg	2.5 mg	2.1 mg
Areal capacitance (20 mV s^−1^)	120.8 mF cm^−2^	249.6 mF cm^−2^	151.8 mF cm^−2^
Mass capacitance (20 mV s^−1^)	120.8 F g^−1^	99.8 F g^−1^	72.3 F g^−1^

## Data Availability

Data are contained within the article.

## Supplementary Materials

The supporting information can be downloaded at: https://www.mdpi.com/article/10.3390/nano14100887/s1, Text S1. Experimental section. Figure S1: The CV and GCD curves for the Ti_3_C_2_T_x_/NF-1.0, Ti_3_C_2_T_x_/NF-2.0, and Ti_3_C_2_T_x_/NF-3.0 electrodes at a series of scan rates and current densities; Figure S2: The b values of the Ti_3_C_2_T_x_/NF-1.0, Ti_3_C_2_T_x_/NF-2.0, and Ti_3_C_2_T_x_/NF-3.0 electrodes; Figure S3: CV partition analysis of Ti_3_C_2_T_x_/NF-1.0 and Ti_3_C_2_T_x_/NF-3.0 electrodes showing the capacitive contribution to the total current at select scan rates of 30 and 100 mV s^−1^; Figure S4. The CV and GCD curves of symmetric supercapacitor device; Figure S5. The *b*-value of symmetric supercapacitor device; Figure S6. Ragone plots of this work compared with previously reported devices.
