# Supplementary material for: Construction of Monolayer Ti3C2Tx MXene on Nickel Foam under High Electrostatic Fields for High-Performance Supercapacitors
Source: Nanomaterials (Basel). 2024 May 19;14(10):887. doi: 10.3390/nano14100887 (PMC11124477; doi:10.3390/nano14100887)
Supplement: Supplementary file 1 [file nanomaterials-14-00887-s001.zip › nanomaterials-2984436-supplementary.pdf]

Supporting Information

The construction of monolayer $Ti_3C_2T_x$ MXene on nickel foam under high electrostatic fields for high-performance supercapacitors

Liyong Zhang[1], Jijie Chen[1], Guanzhi Wei[1], Han Li[2,*], Guanbo Wang[3], Tongjie Li[1], Juan Wang[1], Yehu Jiang[4], Le Bao[5] and Yongxing Zhang[2,*]

[1]Department of Mechanical Engineering, Anhui Science and Technology University, Chuzhou City 235000, People's Republic of China

[2]Anhui Province Key Laboratory of Pollutant Sensitive Materials and Environmental Remediation, Anhui Province Key Laboratory of Intelligent Computing and Applications, Anhui Province Industrial Generic Technology Research Center for Alumics Materials, Huaibei Normal University, Huaibei 235000, People's Republic of China

[3]Jiangsu Zhonggong High-End Equipment Research Institute Co., Ltd. Taizhou City 235000, People's Republic of China

[4]Anhui Zhongxin Technology Co., Ltd. Chuzhou City 235000, People's Republic of China

[5]Department of Mechatronics Engineering, Hanyang University, Ansan 15588, South Korea

[*]Correspondence: lh2017@mail.ustc.edu.cn; zyx07157@mail.ustc.edu.cn

List of Texts and Figures

Text S1. Experimental section.

Figure S1. The CV and GCD curves for $Ti_3C_2T_x$/NF-1.0, $Ti_3C_2T_x$/NF-2.0 and $Ti_3C_2T_x$/NF-3.0 electrode at a series of scan rates and current densities.

Figure S2. The b value of $Ti_3C_2T_x$/NF-1.0 and $Ti_3C_2T_x$/NF-3.0 electrode.

Figure S3. CV partition analysis of $Ti_3C_2T_x$/NF-1.0 and $Ti_3C_2T_x$/NF-3.0 electrode showing the capacitive contribution to the total current at select scan rates of 30 and 100 mV s^{-1}.

Figure S4. The CV and GCD curves of symmetric supercapacitor device.

Figure S5. The b-value of symmetric supercapacitor device.

Figure S6. Ragone plots of this work compared with previously reported devices.

Text S1. Experimental section

Chemicals: Powder of Al (99.9%; -325 mesh) were purchased from Alfa Aesar. Powder of Ti (99.9%; -200 mesh) and HF were purchased from Aladdin. Powder of C, anhydrous sodium sulfatewere (Na_2SO_4) and potassium chloride (KCl) s were purchased from Sinopharm Chemical Reagent Co. Ltd. (Shanghai, China). All chemicals were used without any further purification.

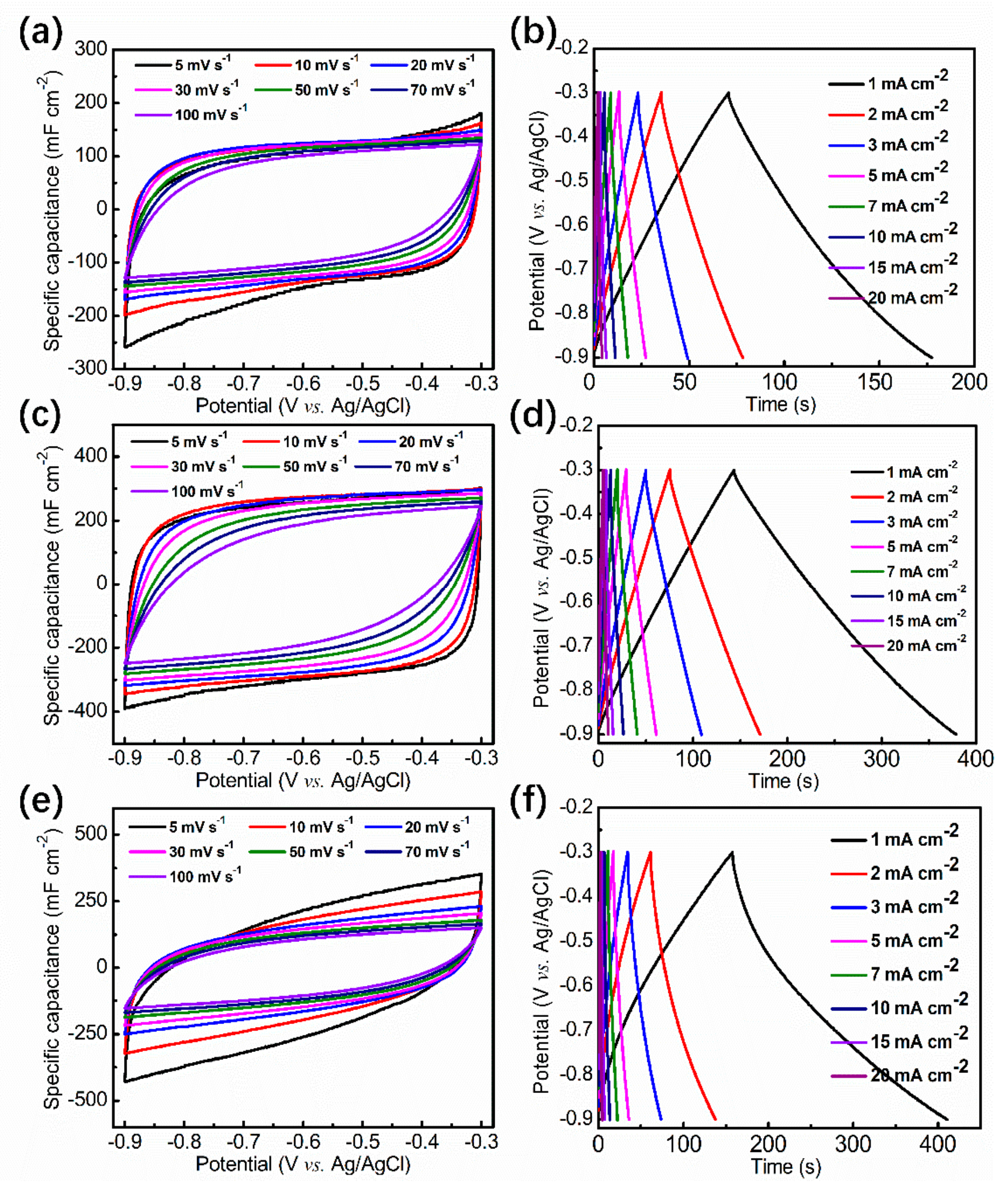

Figure S1. The CV and GCD curves for $Ti_3C_2T_x$/NF-1.0, $Ti_3C_2T_x$/NF-2.0 and $Ti_3C_2T_x$/NF-3.0 electrode at a series of scan rates and current densities.

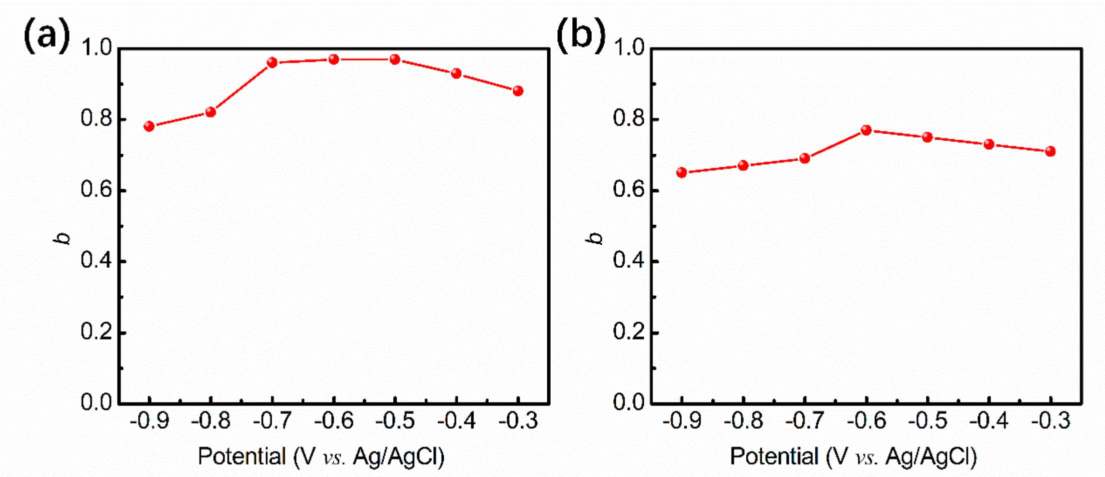

Figure S2. The b value of $Ti_3C_2T_x$/NF-1.0 and $Ti_3C_2T_x$/NF-3.0 electrode.

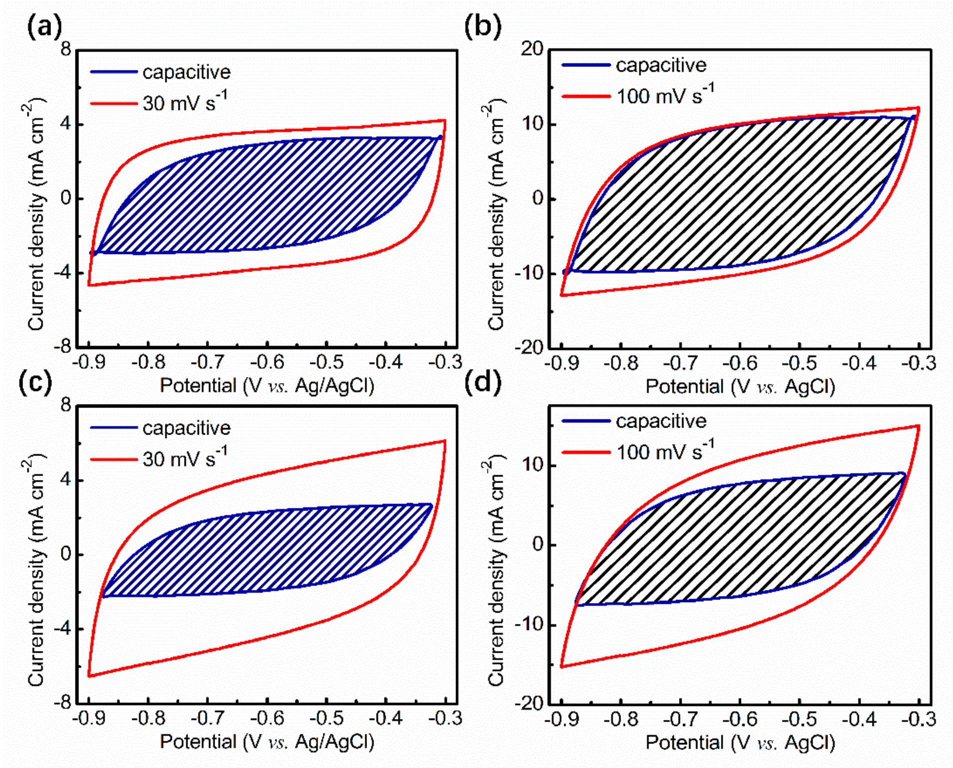

Figure S3. CV partition analysis of $Ti_3C_2T_x$/NF-1.0 and $Ti_3C_2T_x$/NF-3.0 electrode showing the capacitive contribution to the total current at select scan rates of 30 and 100 mV s^{-1}.

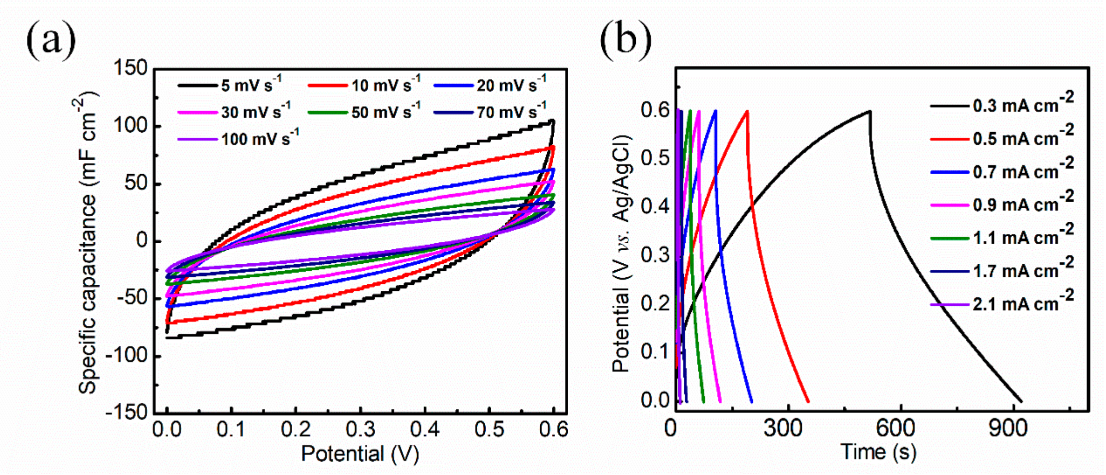

Figure S4. The CV and GCD curves of symmetric supercapacitor device.

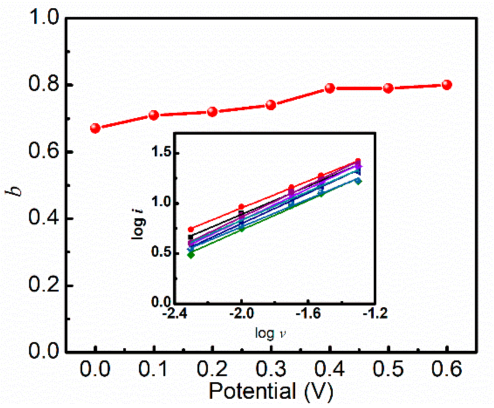

Figure S5. The b-value of symmetric supercapacitor device.

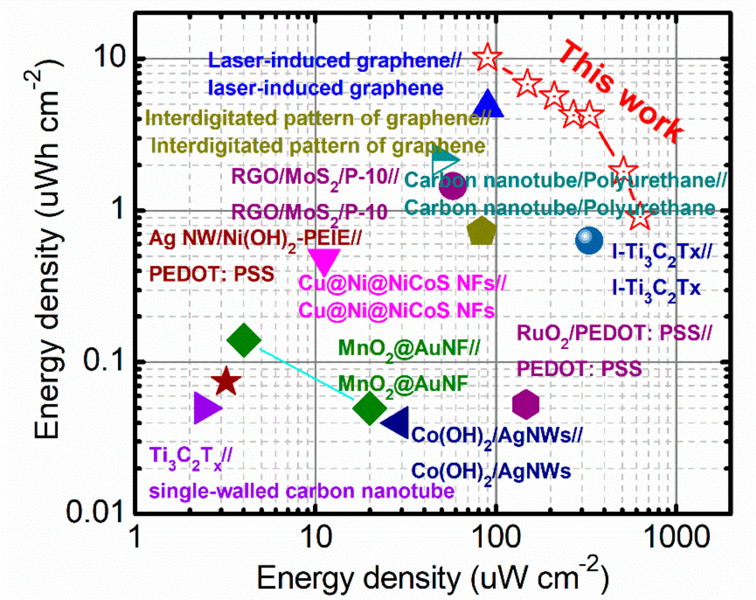

Figure S6. Ragone plots of this work compared with previously reported devices.
